# Global, regional, and national burden of interstitial lung diseases and pulmonary sarcoidosis from 2000 to 2021: a systematic analysis of incidence, mortality, and disability-adjusted life years

**DOI:** 10.3389/fpubh.2025.1578480

**Published:** 2025-06-16

**Authors:** Mi Zhou, Yazhe Zhou, Xin Yang, Kaizhuo Zhou, Xin Zhu

**Affiliations:** ^1^Department of Respiratory and Critical Care Medicine, The First Affiliated Hospital of Chongqing Medical University, Chongqing, China; ^2^The First Clinical College of Chongqing Medical University, Chongqing, China; ^3^Department of Urology, The First Affiliated Hospital of Chongqing Medical University, Chongqing, China

**Keywords:** interstitial lung disease, pulmonary sarcoidosis, incidence, mortality, disability-adjusted life years, aging

## Abstract

**Objectives:**

Interstitial lung diseases (ILDs) and pulmonary sarcoidosis represent a group of disorders characterized by diffuse parenchymal lung damage and chronic inflammation, leading to impaired lung function and gas exchange. These conditions may ultimately result in progressive respiratory failure and increased mortality. This study aimed to assess the global, regional, and national burden of ILDs and pulmonary sarcoidosis.

**Methods:**

Data from the Global Burden of Disease (GBD) 2021 database were used to analyze the incidence, mortality, and disability-adjusted life years (DALYs) associated with ILDs and pulmonary sarcoidosis from 2000 to 2021. Temporal trends were evaluated using Joinpoint regression analysis, and the relationship between disease burden and the sociodemographic index (SDI) was explored by stratifying the data into five SDI categories. Age-standardized rates were calculated to adjust for differences in population structure, and data visualization was performed using R software.

**Results:**

Between 2000 and 2021, the global burden of ILDs and pulmonary sarcoidosis increased, as reflected by rising age-standardized incidence rates (ASIR), mortality rates (ASMR), and disability-adjusted life years rates (ASDR). Older adults, particularly those aged 75 years and above, bore the highest burden. High-SDI regions exhibited higher ASIR, likely due to better diagnostic capabilities, while low-SDI regions experienced higher ASMR and ASDR, potentially due to limited access to healthcare. Gender differences were observed, with males generally having higher rates than females.

**Conclusions:**

Despite advances in diagnostics and treatment over recent decades, the global burden of ILDs and pulmonary sarcoidosis remains substantial, with marked disparities across age groups, genders, and SDI regions. Understanding these epidemiological patterns is essential for developing effective prevention and management strategies.

## Background

Interstitial lung diseases (ILDs) represent a heterogeneous group of over 200 parenchymal lung disorders, with etiologies ranging from identifiable causes, such as environmental exposures and autoimmune diseases, to idiopathic origins (1, 2). Among these, idiopathic pulmonary fibrosis (IPF) is the most common form of ILDs with an unknown etiology. It is a chronic, irreversibly progressive fibrotic lung disease associated with a poor prognosis (3, 4). In recent years, the global incidence of IPF has been rising, with multiple countries reporting increasing trends (5–7). For instance, in the United Kingdom, the incidence of IPF has been estimated to grow by ~5% annually (8). ILDs, particularly those with progressive pulmonary fibrosis (PPF) phenotype, have a profound impact on patients' quality of life and survival (9). The concept of PPF has recently gained growing attention, highlighting shared clinical and pathological characteristics across various ILDs, including advancing fibrosis, respiratory deterioration, and an increased risk of mortality, regardless of the underlying cause (10, 11).

In addition to ILDs, pulmonary sarcoidosis is a granulomatous disorder with highly variable and often unpredictable clinical manifestations (12). While many cases resolve spontaneously or remain stable, a subset of patients progresses to chronic disease with significant fibrosis, leading to substantial morbidity and mortality. The overlap between fibrotic sarcoidosis and other progressive ILDs poses additional challenges for diagnosis and management, underscoring the need to understand both disease-specific and shared mechanisms of progression (13, 14). Given the clinical, pathological, and epidemiological overlaps, the Global Burden of Disease (GBD) framework often categorizes ILDs and pulmonary sarcoidosis together.

Previous studies using GBD 2017 and 2019 data have provided valuable insights into the global and regional burden of ILDs and pulmonary sarcoidosis, with analyses covering the periods 1990–2017 or 1990–2019, respectively (15–17). These works collectively showed an increasing prevalence and significant variations in disease burden and outcomes among different regions and socio-demographic index (SDI) levels. An updated analysis further demonstrated that, except for low-middle SDI regions, the age-standardized prevalence rates (ASPR) significantly increased across all SDI quintiles from 1990 to 2019 (16). Additionally, findings based on the GBD 2019 indicated that high-SDI regions exhibited the highest ASPR, while low-middle SDI regions recorded the highest age-standardized mortality rate (ASMR) and age-standardized DALY rate (ASDR), followed closely by low-SDI regions (17). These data emphasize that socioeconomic development, as measured by SDI, plays a crucial role in shaping the geographic and temporal distribution of disease burden. Additionally, most studies report that the burden of ILDs and pulmonary sarcoidosis increases markedly with age, particularly in individuals over 75 years, and is generally higher in males than in females, partially due to a combination of biological, occupational, and lifestyle factors (15, 18).

To our knowledge, this is the first study to provide a systematic epidemiological analysis of ILDs and pulmonary sarcoidosis using the latest GBD 2021 data, focusing on the period from 2000 to 2021. This period is characterized by major shifts in diagnostic techniques and criteria, which may affect temporal and spatial trends of disease burden (18). Our work expands upon previous research by incorporating the most recent, comprehensive estimates and exploring in detail the influence of socio-demographic index (SDI), age, and gender on disease burden worldwide.

## Methods

### Data availability

This study utilized data from the GBD 2021 database, which provides a comprehensive assessment of health risks associated with 371 diseases and injuries, as well as 88 risk factors, across 204 countries and territories and 811 subnational regions (14). Data specific to ILDs and pulmonary sarcoidosis including incidence, mortality, and DALYs ranging from 2000 to 2021 were abstracted via the Global Health Data Exchange platform (http://ghdx.healthdata.org/gbd-results-tool). To analyze the age distribution of disease burden in ILDs and pulmonary sarcoidosis, patients were grouped into four age categories (0–14, 15–49, 50–74, and ≥75 years) according to the standard classifications of the GBD database. This approach ensures consistency, comparability, and clear differentiation among children, young and middle-aged adults, older adults, and oldest adults. The start year of 2000 was chosen for analysis to reflect the widespread global adoption of HRCT in the diagnosis of ILDs and the establishment of consistent, international diagnostic criteria (19–21). Notably, the recent development of the interstitial lung disease imaging-reporting and data system (ILD-RADS) has demonstrated significant utility and reproducibility, offering the potential to standardize HRCT reporting and improve diagnostic accuracy for ILDs (20). By restricting our analysis to 2000–2021, we ensured improved data comparability across countries and minimized biases arising from changes in diagnostic sensitivity. All disease burden metrics, age group stratifications, and trend analyses were aligned with GBD 2021 conventions to facilitate consistency.

### Disease burden and temporal trend analysis

To investigate the global distribution and regional disparities in the burden of ILDs and pulmonary sarcoidosis, global maps and regional comparative analyses were generated. Visualization was conducted using the R software package (version 4.2.3) and JD_GBDR (V2.24, Jingding Medical Technology Co., Ltd.), employing the “ggplot2” and “sf” packages to create maps illustrating the disease burden.

In addition, temporal trends of ILDs and pulmonary sarcoidosis from 2000 to 2021 were analyzed using Joinpoint regression analysis. This analysis was performed with the “segment” and “broom” R packages to identify significant temporal changes. The age-standardized average annual percentage change (APC) and estimated annual percentage change (EAPC) were calculated using Joinpoint regression and linear regression models, respectively. Statistical significance for APC and EAPC was assessed with 95% confidence intervals (CIs) (22–24). For GBD-derived metrics such as incidence, mortality, and DALYs, we reported 95% uncertainty intervals (UIs) according to GBD study conventions, reflecting both sampling and model-related uncertainty.

### Sociodemographic index (SDI) analysis

SDI, a composite measure incorporating fertility rates, educational attainment, and per capita income, was utilized to represent the overall level of social and economic development in different countries (14). The association between SDI and the burden of ILDs and pulmonary sarcoidosis was assessed by stratifying countries and territories into five SDI categories: low (0–0.46), low-middle (0.46–0.61), middle (0.61–0.69), high-middle (0.69–0.81), and high (0.81–1.00). According to data from GBD 2021, the 204 countries and territories included in the study were classified based on these SDI intervals (25). This stratification facilitated comparisons of disease burden across varying levels of socioeconomic development. Data processing and visualization were performed using the “dplyr” (version 1.1.3) and “ggplot2” packages in R.

### Statistics

The burden of ILDs and pulmonary sarcoidosis was evaluated using key metrics, including incidence, mortality, and DALYs numbers, along with their corresponding rates per 100,000 population. Total numbers of incidence, mortality, and DALYs were calculated based on the all-age population. To account for variations in age structures across populations, age-standardized incidence rates (ASIR), ASMR, and ASDR were calculated using the direct method of standardization based on age-standardized populations, with the GBD world standard population employed as the reference (26). This study adhered to the Strengthening the Reporting of Observational Studies in Epidemiology (STROBE) guidelines to ensure methodological rigor and transparency (27).

## Results

### Global trends

#### Incidence

In 2000, there were 213,044 incident cases (95% UI: 185,864 to 242,213) of ILDs and pulmonary sarcoidosis, with an ASIR of 4.16 cases per 100,000 population (95% UI: 3.64 to 4.68). By 2010, the incident cases increased to 300,766 (95% UI: 265,033 to 335,779), and ASIR rose to 4.63 (95% UI: 4.08 to 5.16). In 2021, the incident cases further rose to 390,267 (95% UI: 346,393 to 433,403), while ASIR slightly decreased to 4.54 (95% UI: 4.05 to 5.04) compared with 2010. Between 2000 and 2010, the ASIR increased by 11.27% (95% UI: 9.16 to 13.55). From 2010 to 2021, ASIR exhibited a slight decline, with a rate change of −1.83% (95% UI: −3.64 to −0.09). Over the entire period (2000–2021), the EAPC in ASIR was 0.47% (95% CI: 0.31 to 0.62), reflecting a modest overall increase in the global incidence rate (Table 1).

**Table 1:** Incidence of interstitial lung diseases and pulmonary sarcoidosis between 2000 and 2021.

**Location**	**2000**		**2010**		**2021**		**2000–2010**	**2010–2021**	**2000–2021**
	**Incident cases**	**Incidence rate**	**Incident cases**	**Incidence rate**	**Incident cases**	**Incidence rate**	**Rate change**	**Rate change**	**EAPC**
Global	213,043.84 (185,863.85, 242,213.47)	4.16 (3.64, 4.68)	300,766.40 (265,033.02, 335,779.32)	4.63 (4.08, 5.16)	390,267.11 (346,393.42, 433,403.27)	4.54 (4.05, 5.04)	11.27 (9.16, 13.55)	−1.83 (−3.64, −0.09)	0.47 (0.31, 0.62)
East Asia	24,129.66 (20,187.19, 28,959.38)	1.87 (1.58, 2.20)	41,587.73 (35,614.76, 49,103.96)	2.53 (2.17, 2.95)	50,030.89 (42,927.49, 57,599.83)	2.31 (2.02, 2.64)	35.39 (27.63, 43.66)	−8.57 (−11.43, −5.67)	1.31 (0.76, 1.86)
Southeast Asia	5,419.69 (4,586.65, 6,327.66)	1.46 (1.25, 1.68)	7,613.88 (6,538.60, 8,830.97)	1.53 (1.32, 1.75)	11,245.44 (9,856.84, 12,737.72)	1.62 (1.42, 1.81)	4.83 (3.72, 5.82)	5.83 (2.70, 10.19)	0.53 (0.51, 0.55)
Oceania	206.41 (185.09, 231.29)	3.82 (3.48, 4.20)	284.93 (255.95, 318.68)	3.97 (3.61, 4.36)	419.30 (384.88, 456.21)	4.20 (3.91, 4.51)	3.77 (1.51, 6.05)	5.92 (1.50, 10.11)	0.47 (0.45, 0.49)
Central Asia	1,675.86 (1,515.67, 1,866.33)	3.00 (2.74, 3.28)	1,936.40 (1,749.00, 2,147.14)	2.93 (2.66, 3.21)	2,848.59 (2,621.60, 3,093.58)	3.38 (3.12, 3.65)	−2.34 (−4.01, −0.75)	15.48 (12.49, 18.31)	0.79 (0.57, 1.01)
Central Europe	3,492.43 (3,121.23, 3,931.57)	2.36 (2.10, 2.66)	3,908.38 (3,507.93, 4,351.74)	2.48 (2.21, 2.80)	4,033.56 (3,661.38, 4,442.55)	2.42 (2.20, 2.69)	5.00 (3.90, 6.17)	−2.27 (−4.72, 0.36)	0.16 (0.05, 0.27)
Eastern Europe	4,425.24 (3,764.22, 5,207.71)	1.70 (1.45, 1.98)	3,234.24 (2,698.83, 3,790.87)	1.24 (1.04, 1.46)	2,682.32 (2,299.15, 3,096.10)	1.04 (0.89, 1.21)	−27.40 (−29.73, −25.14)	−15.92 (−18.63, −13.07)	−2.44 (−2.66, −2.23)
High -income Asia Pacific	2,8651.83 (24,229.37, 33,511.38)	10.98 (9.35, 12.73)	39,513.23 (34,105.99, 45,634.04)	12.26 (10.67, 13.98)	43,787.31 (38,399.14, 49,649.78)	11.59 (10.25, 13.02)	11.67 (7.91, 15.70)	−5.43 (−7.85, −2.96)	0.18 (−0.03, 0.39)
Australasia	1,383.02 (1,263.72, 1,517.39)	4.73 (4.33, 5.17)	2,283.88 (2,082.99, 2,492.43)	6.04 (5.51, 6.61)	3,392.34 (3,070.03, 3,718.02)	6.45 (5.88, 7.02)	27.74 (23.62, 31.69)	6.80 (3.59, 9.97)	1.44 (1.19, 1.69)
Western Europe	25,505.89 (23,046.40, 28,009.96)	4.30 (3.89, 4.71)	36,739.24 (33,238.86, 40,508.03)	5.27 (4.78, 5.77)	43,619.93 (39,614.12, 47,748.76)	5.30 (4.82, 5.81)	22.57 (20.39, 24.81)	0.63 (−0.83, 2.03)	0.97 (0.71, 1.22)
Southern Latin America	4,323.40 (4,017.65, 4,663.65)	7.76 (7.21, 8.36)	6,255.56 (5,830.56, 6,700.20)	9.15 (8.54, 9.81)	8,476.90 (7,913.38, 9,062.32)	9.90 (9.26, 10.57)	17.89 (14.96, 20.66)	8.22 (5.49, 10.64)	1.17 (1.05, 1.29)
High -income North America	40,137.12 (35,055.04, 45,663.47)	10.15 (8.85, 11.49)	49,290.20 (44,299.33, 54,191.46)	10.30 (9.30, 11.31)	66,609.12 (58,422.82, 75,152.76)	10.95 (9.73, 12.20)	1.46 (−4.25, 7.31)	6.39 (3.16, 9.44)	0.33 (0.26, 0.41)
Caribbean	561.72 (497.61, 634.49)	1.59 (1.42, 1.78)	791.12 (707.28, 882.06)	1.83 (1.63, 2.03)	1,003.14 (917.01, 1,094.45)	1.89 (1.73, 2.07)	14.52 (12.66, 16.47)	3.72 (1.19, 6.64)	0.83 (0.70, 0.97)
Andean Latin America	3,835.07 (3,554.20, 4,107.89)	13.73 (12.67, 14.75)	7,429.47 (6,932.19, 7,932.41)	18.32 (17.09, 19.61)	11,837.69 (11,093.94, 12,531.27)	20.47 (19.15, 21.69)	33.44 (30.18, 36.81)	11.69 (9.14, 14.54)	2.11 (1.82, 2.40)
Central Latin America	5,372.65 (4,752.22, 6,047.71)	4.18 (3.69, 4.70)	8,311.91 (7,411.56, 9,230.42)	4.64 (4.14, 5.16)	12,172.27 (10,942.78, 13,423.36)	4.81 (4.33, 5.29)	10.99 (8.19, 13.31)	3.54 (2.17, 5.03)	0.68 (0.59, 0.78)
Tropical Latin America	3,481.50 (3,039.97, 3,949.44)	2.59 (2.25, 2.92)	4,478.34 (3,945.62, 5,035.68)	2.48 (2.17, 2.79)	6,639.97 (5,821.18, 7,441.33)	2.62 (2.29, 2.94)	−4.42 (−7.27, −1.66)	5.71 (3.65, 7.55)	0.05 (−0.09, 0.18)
North Africa and Middle East	6,367.41 (5,516.84, 7,354.46)	2.36 (2.06, 2.66)	10,214.15 (8,937.39, 11,657.08)	2.70 (2.39, 3.03)	15,113.49 (13,573.57, 16,830.54)	2.85 (2.58, 3.15)	14.35 (12.52, 16.48)	5.58 (2.64, 8.98)	0.92 (0.81, 1.04)
South Asia	48,125.11 (40,930.01, 55,574.93)	6.17 (5.26, 7.09)	69,375.61 (59,331.42, 80,008.63)	6.44 (5.48, 7.43)	96,281.45 (84,137.37, 108,635.45)	6.44 (5.65, 7.26)	4.27 (3.51, 5.07)	0.05 (−3.34, 4.21)	0.25 (0.19, 0.31)
Central Sub-Saharan Africa	593.36 (500.99, 694.67)	1.78 (1.51, 2.04)	837.60 (706.46, 983.14)	1.82 (1.57, 2.08)	1,271.70 (1,103.24, 1,454.64)	1.88 (1.66, 2.10)	2.52 (0.38, 4.75)	3.17 (−0.90, 7.94)	0.29 (0.28, 0.31)
Eastern Sub-Saharan Africa	1,698.00 (1,414.53, 1,998.91)	1.53 (1.30, 1.74)	2,253.82 (1,884.89, 2,649.26)	1.52 (1.30, 1.74)	3,243.79 (2,779.59, 3,752.94)	1.53 (1.34, 1.72)	−0.46 (−1.33, 0.46)	0.60 (−2.39, 4.25)	0.02 (0.00, 0.03)
Southern Sub-Saharan Africa	1,954.33 (1,671.85, 2,256.99)	5.06 (4.33, 5.82)	2,251.42 (1,923.93, 2,601.17)	4.74 (4.05, 5.43)	2,577.13 (2,241.39, 2,928.29)	4.21 (3.69, 4.73)	−6.31 (−7.45, −5.11)	−11.23 (−14.07, v7.75)	−1.07 (−1.16, −0.97)
Western Sub-Saharan Africa	1,704.15 (1,414.56, 2,006.67)	1.24 (1.06, 1.42)	2,175.28 (1,795.77, 2,580.81)	1.17 (1.00, 1.35)	2,980.76 (2,544.17, 3,485.63)	1.13 (0.99, 1.28)	−5.70 (−6.99, −4.42)	−3.24 (−5.99, 0.31)	−0.40 (−0.44, −0.36)
High-middle SDI	32,290.06 (28,528.78, 36,726.76)	2.62 (2.33, 2.95)	46,835.21 (41,740.82, 52,572.19)	3.12 (2.78, 3.47)	56,001.46 (50,154.74, 61,921.28)	2.97 (2.68, 3.27)	19.00 (15.66, 22.63)	−4.75 (−7.06, −2.80)	0.66 (0.37, 0.94)
High SDI	92,066.88 (80,627.15, 104,059.69)	7.35 (6.45, 8.29)	123,508.89 (110,303.49, 137,094.62)	8.05 (7.19, 8.92)	155,237.81 (137,458.20, 174,122.24)	8.19 (7.29, 9.07)	9.59 (5.76, 13.28)	1.68 (0.27, 2.97)	0.49 (0.40, 0.59)
Low -middle SDI	36,863.66 (31,606.79, 42,382.86)	4.52 (3.87, 5.16)	51,644.17 (44,358.50, 59,123.73)	4.78 (4.10, 5.49)	70,990.24 (62,702.07, 79,388.32)	4.85 (4.28, 5.42)	5.93 (5.02, 6.92)	1.34 (−2.02, 5.28)	0.40 (0.34, 0.47)
Low SDI	9,718.66 (8,266.00, 11,267.69)	3.24 (2.77, 3.70)	13,277.25 (11,287.45, 15,418.78)	3.35 (2.88, 3.84)	18,292.09 (16,088.70, 20,678.40)	3.33 (2.95, 3.70)	3.43 (2.11, 4.83)	−0.79 (−4.29, 3.81)	0.11 (0.05, 0.17)
Middle SDI	41,984.57 (36,093.55, 48,466.09)	2.88 (2.51, 3.27)	65,345.97 (57,227.17, 74,493.08)	3.40 (2.98, 3.82)	89,561.47 (79,276.37, 100,011.02)	3.37 (3.00, 3.73)	17.98 (16.02, 20.15)	−0.99 (−3.22, 1.82)	0.87 (0.63, 1.10)

EAPC, estimated annual percentage change; SDI, sociodemographic index; UI, uncertainty interval; CI, confidence interval.

**Figure 1:**
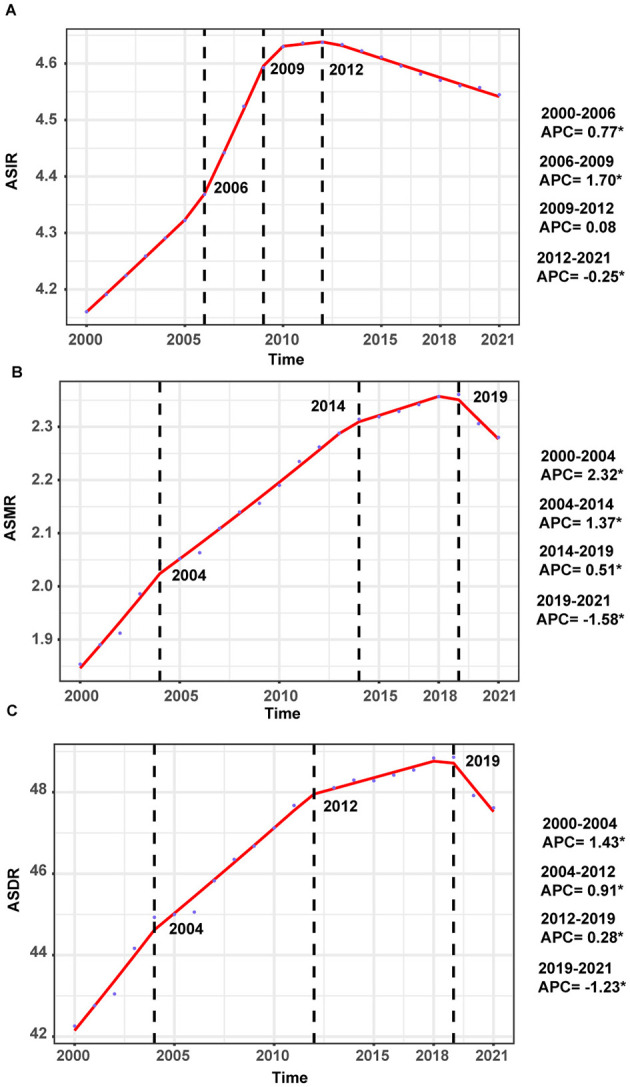
Annual percent change (APC) and trends in the global incidence, mortality, and disability-adjusted life years (DALYs) of interstitial lung diseases (ILDs) and pulmonary sarcoidosis from 2000 to 2021. **(A)** Age-standardized incidence rates (ASIR). **(B)** Age-standardized mortality rates (ASMR). **(C)** Age-standardized DALYs rates (ASDR).

The temporal trends in ASIR of ILDs and pulmonary sarcoidosis from 2000 to 2021 showed a dynamic pattern with four distinct intervals based on APC (Figure 1A). From 2000 to 2006, the ASIR exhibited a moderate increase (APC: 0.77%). This was followed by a sharper rise between 2006 and 2009, where the APC peaked at 1.70%. Subsequently, the trend stabilized from 2009 to 2012, with a minimal APC of 0.08%. However, from 2012 to 2021, the ASIR demonstrated a gradual decline, reflected by a negative APC of −0.25% (Figure 1A). In 2021, the ASIR by region ranged from 1.04 cases per 100,000 population (95% UI: 0.89 to 1.21) in Eastern Europe to 20.47 cases per 100,000 population (95% UI: 19.15 to 21.69) in Andean Latin America (Table 1). In 2000, the regional ASIR ranged from 1.70 cases per 100,000 population (95% UI: 1.45 to 1.98) in Eastern Europe to 13.73 cases per 100,000 population (95% UI: 12.67 to 14.75) in Andean Latin America (Table 1).

**Figure 2:**
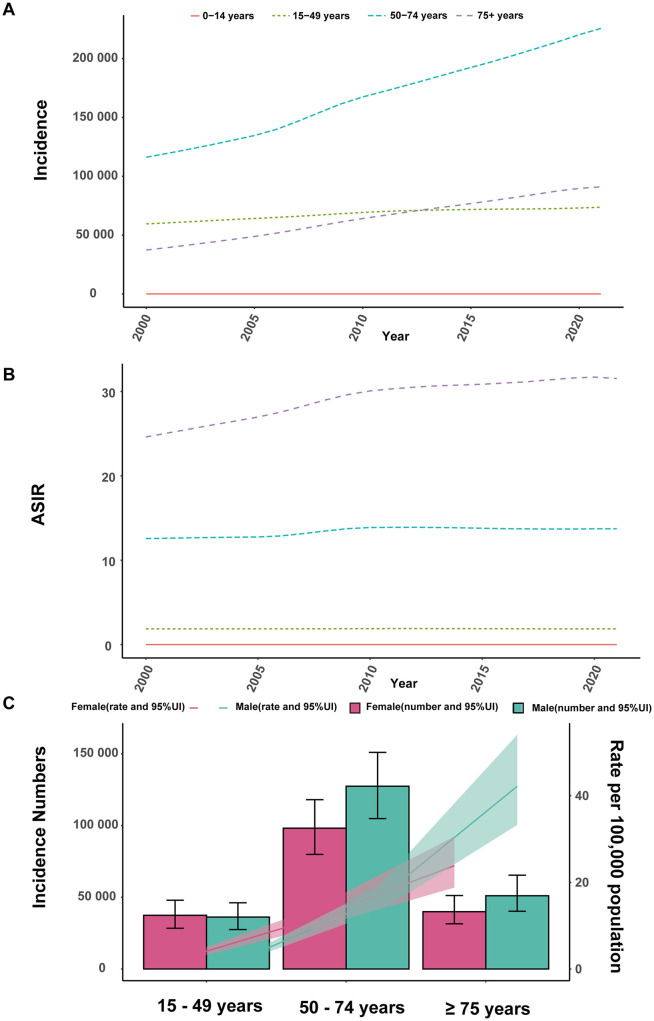
Trends in incidence of interstitial lung diseases (ILDs) and pulmonary sarcoidosis by age and sex, 2000–2021. **(A)** Incidence cases. **(B)** Age-standardized incidence rates (ASIR). **(C)** Incidence cases and ASIR stratified by age and sex.

**Figure 3:**
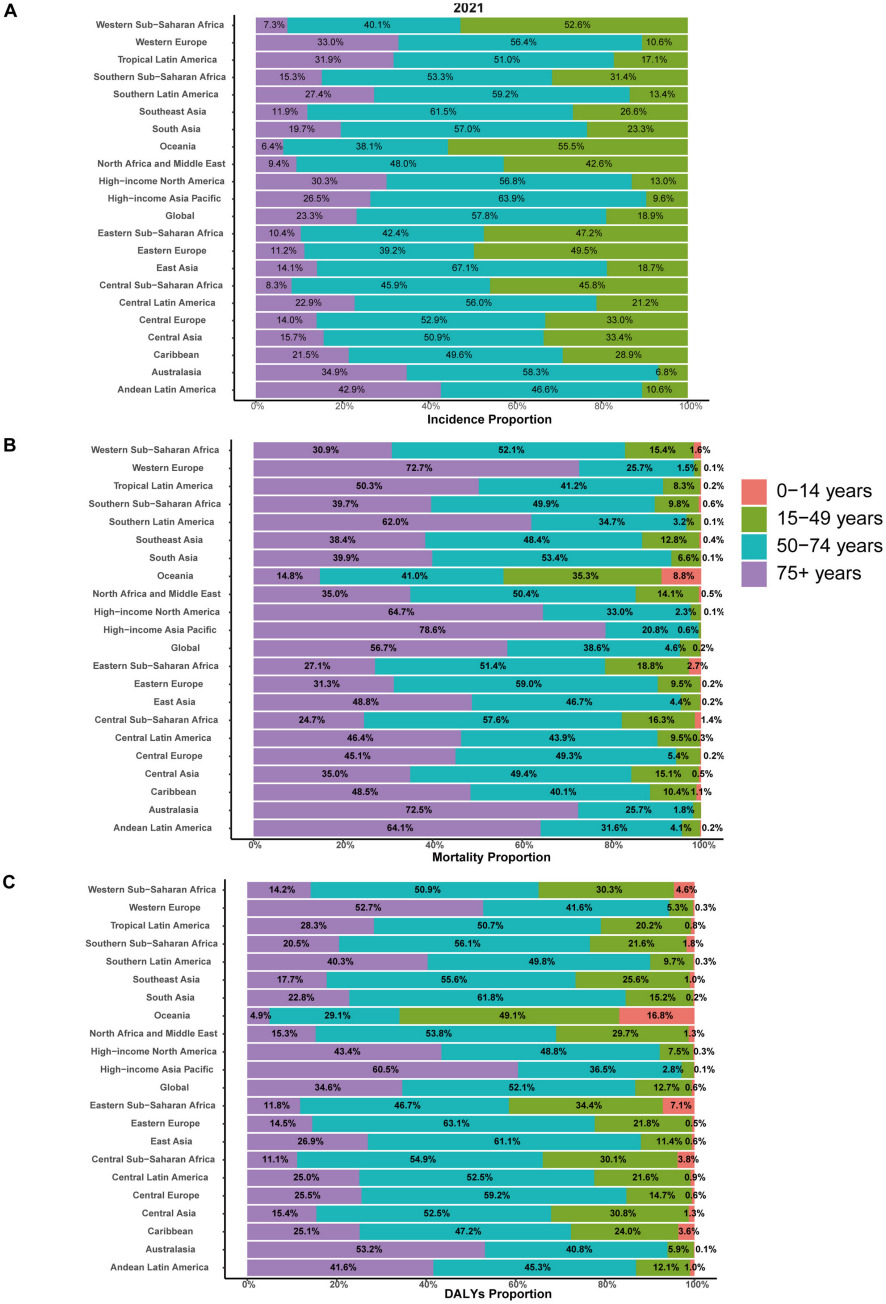
Age-specific distribution of interstitial lung diseases (ILDs) and pulmonary sarcoidosis in terms of incidence, mortality, and disability-adjusted life years (DALYs) in 2021. **(A)** Incidence. **(B)** Mortality. **(C)** DALYs.

Notably, the largest increase of incident cases was observed in individuals aged 50–74 years, comprising 57.8% of all incident cases in 2021. Closely following were individuals aged 75 years and older, comprising 23.3% of all incident cases in the same year (Figures 2A, 3A). The 0–14 years age group was excluded due to negligible incidence and ASIR, and the 15–49 years group had the smallest increase, comprising 18.9% of cases in 2021. Moreover, individuals aged 75 years and older exhibited the highest ASIR consistently and markedly (Figure 2B). Gender differences were also evident, with males generally showing higher ASIR than females across most age groups, particularly in the 50–74 years and 75+ years age groups (Figure 2C).

#### Mortality

In 2000, the global mortality number due to ILDs and pulmonary sarcoidosis was 84,049 (95% UI: 72,114 to 99,247), with an ASMR of 1.85 per 100,000 population (95% UI: 1.60 to 2.17). By 2010, mortality increased to 130,300 (95% UI: 112,610 to 147,936), and the ASMR rose to 2.19 (95% UI: 1.90 to 2.49). In 2021, mortality further rose to 188,222 (95% UI: 161,406 to 212,252), while the ASMR slightly increased to 2.28 (95% UI: 1.96 to 2.56). Between 2000 and 2010, the ASMR increased by 18.14% (95% UI: 12.40 to 24.89). From 2010 to 2021, the ASMR exhibited a slower increase, with a rate change of 4.11% (95% UI: −0.90 to 9.68). Over the entire period (2000–2021), the EAPC in ASMR was 1.12% (95% UI: 0.93 to 1.30) (Supplementary Table 1).

From 2000 to 2004, the global ASMR showed a notable increase, with an APC of 2.32%. This was followed by a continued but slower rise between 2004 and 2014, where the APC decreased to 1.37%. From 2014 to 2019, the trend further stabilized, with a minimal APC of 0.51%. However, from 2019 to 2021, the ASMR demonstrated a decline, reflected by a negative APC of −1.58% (Figure 1B).

Individuals aged 50–74 years and 75+ years contributed to most mortality, with the highest age-standardized mortality rate (ASMR) observed in the 75+ age group from 2000 to 2021, accounting for 56.7% of all mortality cases in 2021 (Figure 3B). Moreover, the 75+ age group exhibits a clear upward trend in absolute mortality numbers over time, with ASMR significantly exceeding those of other age groups. The 50–74 age group shows moderate mortality cases and ASMR, which are higher than those of younger cohorts but substantially lower than the 75+ years group. Gender differences were evident, with males consistently showing higher ASMR and mortality numbers than females across all age groups, particularly in the 50–74 years and 75+ years age groups (Supplementary Figure 1).

#### DALYs

In 2000, the global burden was estimated at approximately 2,095,007 DALYs (95% UI: 1,780,250 to 2,483,987), corresponding to an ASDR of 42.26 per 100,000 population (95% UI: 36.24 to 49.78). By 2021, the global DALYs had increased to 4,042,150 (95% UI: 3,489,795 to 4,516,883), with a slightly higher ASDR of 47.62 per 100,000 population (95% UI: 41.26 to 53.16). The EAPC over the study period was 0.65% (95% CI: 0.51 to 0.78), reflecting a relatively stable trend (Supplementary Table 2). As illustrated in Figure 1C, between 2000 and 2004, the ASDR showed a moderate increase, with an APC of 1.43%. This was followed by a continued but slower rise from 2004 to 2012, during which the APC decreased to 0.91%. From 2012 to 2019, the trend stabilized further, with a minimal APC of 0.28%. However, a notable shift occurred between 2019 and 2021, as the ASDR declined, reflected by a negative APC of −1.23%. In 2021, for the global population, individuals aged 50–74 years accounted for the largest proportion of DALYs (52.1%), followed by the 75+ age group, which contributed 34.6% (Figure 3C). The 75+ age group shows the highest ASDR and a clear upward trend in DALYs from 2000 to 2021. The 50–74 age group also contributes substantially, with relatively stable ASDR but increasing DALYs over time. Males in both groups consistently exhibit higher ASDR and DALYs than females (Supplementary Figure 2).

#### Regional trends by SDI

**Figure 4:**
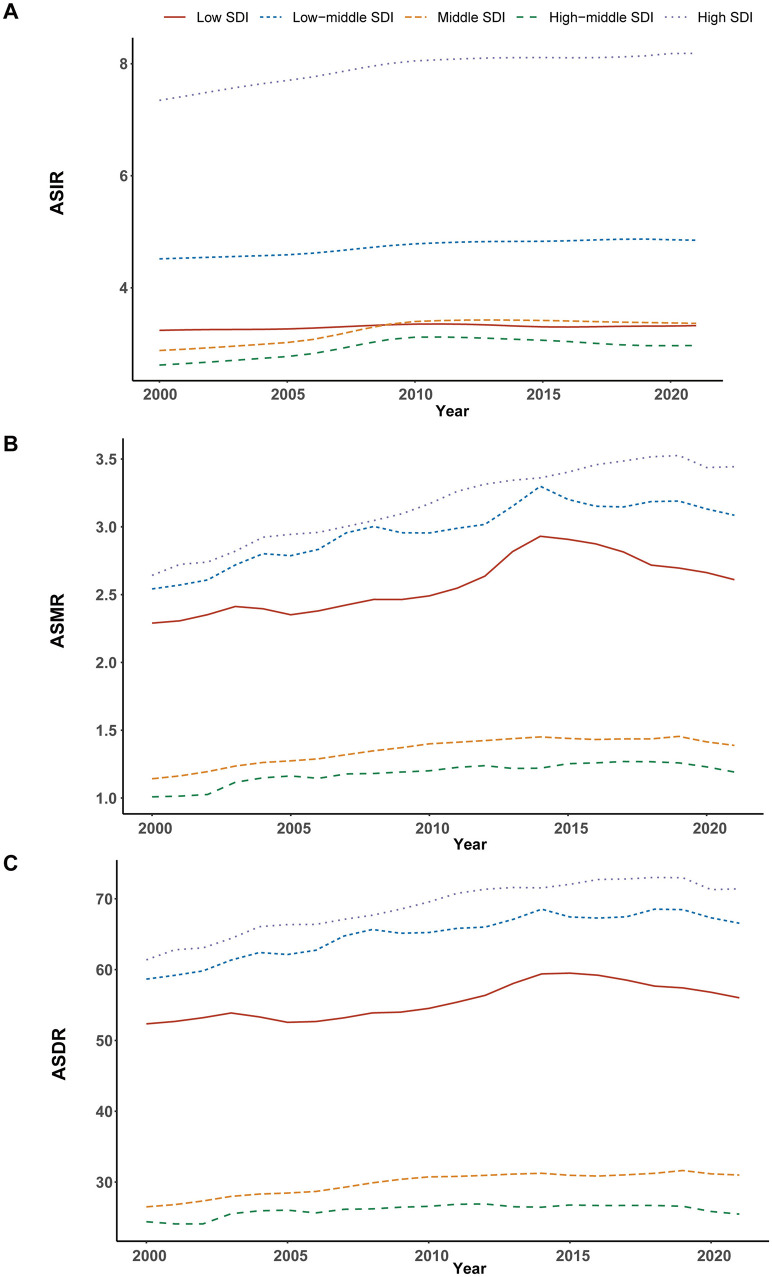
Epidemiologic trends in interstitial lung diseases (ILDs) and pulmonary sarcoidosis in terms of incidence, mortality, and disability-adjusted life years (DALYs) rates across five Sociodemographic Index (SDI) areas from 2000 to 2021. **(A)** Age-standardized incidence rates (ASIR). **(B)** Age-standardized mortality rates (ASMR). **(C)** Age-standardized DALYs rates (ASDR).

**Figure 5:**
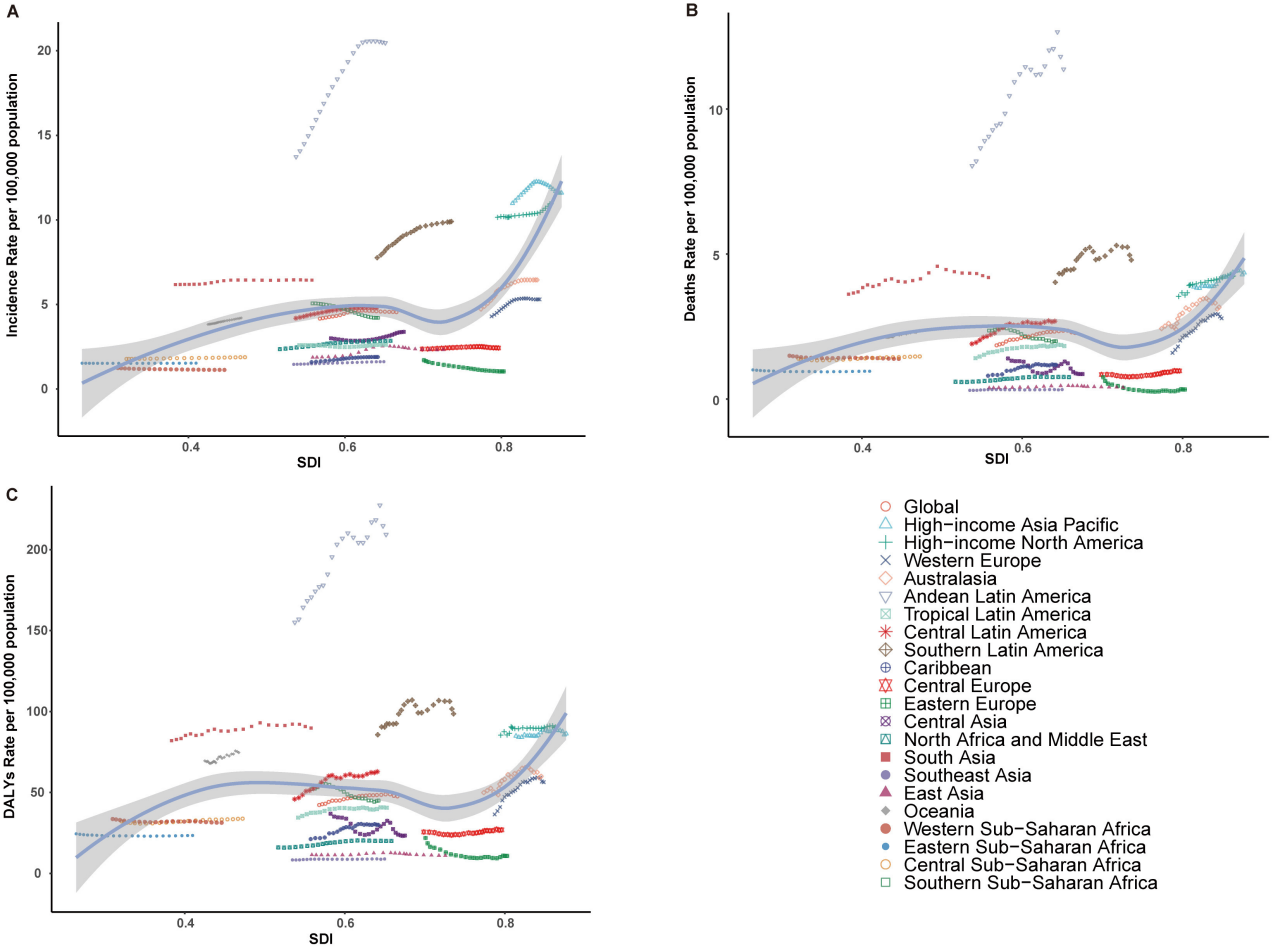
Relationship between the rates of incidence, mortality, and disability-adjusted life years (DALYs) for interstitial lung diseases (ILDs) and pulmonary sarcoidosis, and the regional Sociodemographic Index (SDI) from 2000 to 2021. **(A)** Age-standardized incidence rate (ASIR). **(B)** Age-standardized mortality rate (ASMR). **(C)** Age-standardized DALY rate (ASDR).

From 2000 to 2021, the incidence, mortality, and DALYs associated with ILDs and pulmonary sarcoidosis exhibited distinct trends across different SDI regions. High SDI regions consistently exhibited the highest levels of ASIR, ASMR, and ASDR throughout the study period, with a notable upward trend over the study period. In contrast, high-middle SDI and middle SDI regions consistently show moderate levels of ASIR, ASMR, and ASDR, which are lower than high SDI regions but higher than low and low-middle SDI regions. Meanwhile, low and low-middle SDI regions showed moderate to significant increases in these metrics, particularly in ASMR and ASDR (Table 1; Supplementary Tables 1, 2; Figures 4, 5).

### National trends

#### Incidence

**Figure 6:**
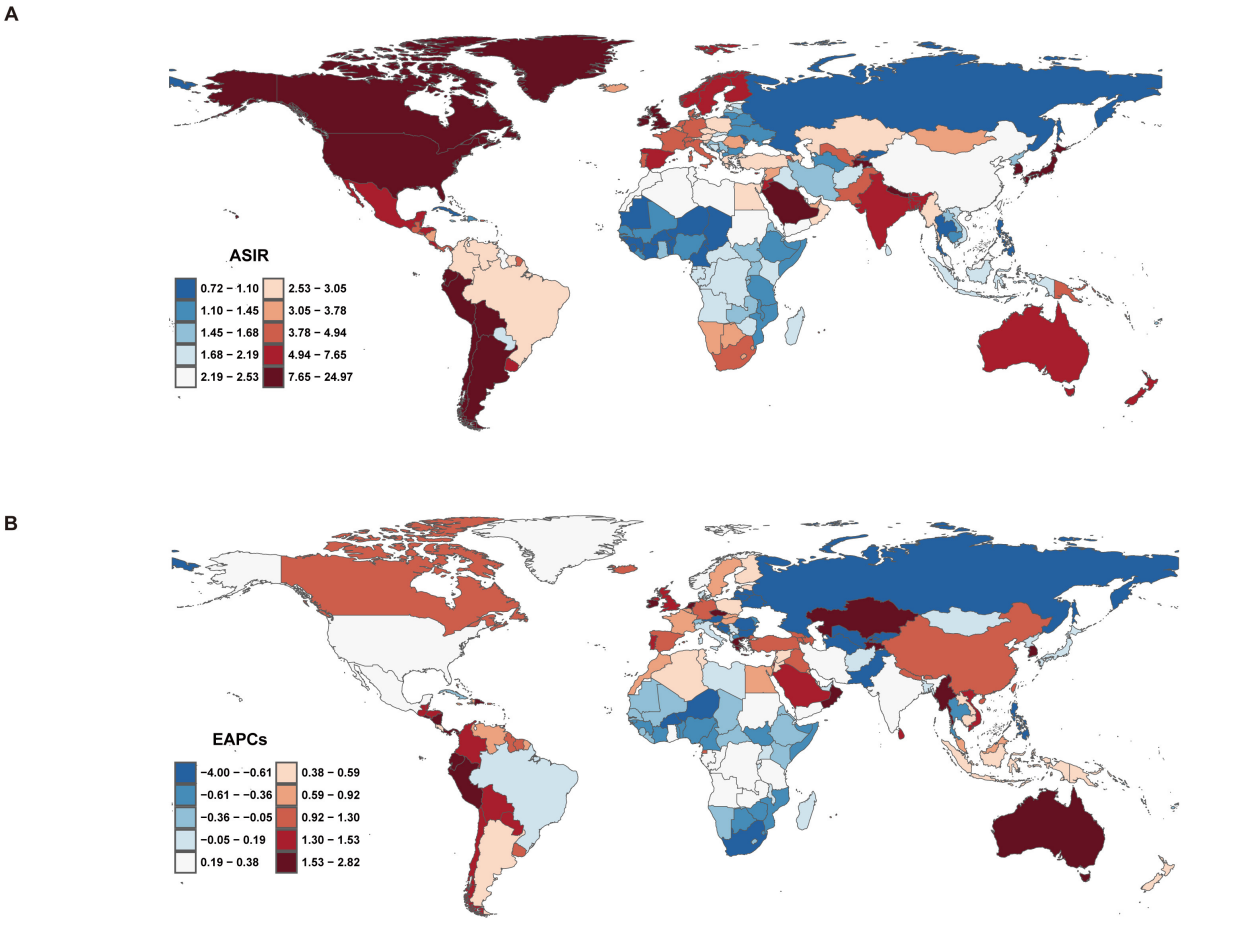
Incidence of interstitial lung diseases (ILDs) and pulmonary sarcoidosis across 204 countries and territories. **(A)** Age-standardized incidence rate (ASIR). **(B)** Estimated annual percentage change (EAPC) in incidence.

In 2021, the global ASIR of ILDs and pulmonary sarcoidosis varied significantly across regions, as shown in the map, which includes data from 204 countries (Figure 6A). The countries with the highest ASIR were Peru (24.73, 95% UI: 23.23 to 26.22), Bolivia (18.48, 95% UI: 16.97 to 20.02), and Chile (15.13, 95% UI: 14.03 to 16.23). In contrast, the countries with the lowest ASIR included the Philippines (0.73, 95% UI: 0.61 to 0.85), Burkina Faso (0.81, 95% UI: 0.70 to 0.93), and Cabo Verde (0.81, 95% UI: 0.70 to 0.93) (Supplementary Table 3). From 2000 to 2020, trends in the EAPC of ASIR also varied widely across regions (Figure 6B). Some regions experienced significant increases in ASIR, with the countries showing the highest EAPC being Ecuador (2.79, 95% UI: 2.39 to 3.20), Kazakhstan (2.78, 95% UI: 2.45 to 3.11), and Greece (2.78, 95% UI: 2.39 to 3.17). In contrast, the countries with the lowest EAPC included Ukraine (−4.04, 95% UI: −4.49 to 3.59), Belarus (−3.23, 95% UI: −3.52 to −2.95), and Latvia (−1.68, 95% UI: −2.14 to −1.22) (Supplementary Table 3).

#### Mortality

Regarding mortality of ILDs and pulmonary sarcoidosis at the national level, in 2021, India recorded the highest mortality number (47,336.08, 95% UI: 30,672.40 to 65,803.94), while Bolivia had the highest ASMR (9.74 per 100,000 people; 95% UI: 6.25 to 14.08). From 2000 to 2020, the countries with the highest EAPC values were Libya (8.97, 95% UI: 7.97 to 9.97), Morocco (6.91, 95% UI: 5.62 to 8.22), and Algeria (6.79, 95% UI: 5.51 to 8.08). In contrast, the countries with the lowest EAPC values included Belarus (−8.49, 95% UI: −9.51 to −7.45), Ukraine (−6.08, 95% UI: −6.93 to −5.23), and Uzbekistan (−5.29, 95% UI: −7.01 to −3.53) (Supplementary Table 4).

#### DALYs

In 2021, India recorded the highest number of DALYs due to ILD and pulmonary sarcoidosis, with 1,124,247.84 cases (95% UI: 750,835.18 to 1,523,498.82). The corresponding ASDR was 95.50 per 100,000 population (95% UI: 63.70 to 129.59). Over the period from 2000 to 2021, India exhibited an EAPC of 0.72 (95% UI: 0.59 to 0.85), indicating a steady increase in the burden of ILD and pulmonary sarcoidosis. The countries with the highest EAPC values from 2000 to 2021 were Libya (4.47, 95% UI: 4.11 to 4.84), Greece (3.99, 95% UI: 2.79 to 5.20), and Guyana (3.98, 95% UI: 3.41 to 4.55). In contrast, the countries with the lowest EAPC values included Belarus (−6.38, 95% UI: −7.12 to −5.64), Ukraine (−5.61, 95% UI: −6.39 to −4.83), and Uzbekistan (−4.73, 95% UI: −6.20 to −3.24) (Supplementary Table 5).

## Discussion

In this study, between 2000 and 2010, the ASIR of ILDs and pulmonary sarcoidosis showed a steady increase, aligned with previous studies that reported a gradual increase in the burden of ILDs (15, 16). The widespread adoption of HRCT beginning in the early 2000s played a pivotal role in improving detection and diagnosis, likely contributing to the observed increase in incidence rates, as more cases were identified and accurately classified (21, 28, 29). However, from 2010 to 2021, the ASIR demonstrated a slight decline, despite a continued increase in absolute case numbers. This shift may reflect a plateau in diagnostic capacity in certain regions, improvements in preventative measures, or changes in environmental and occupational exposures (30, 31). Regionally, high ASIRs were consistently observed in Andean Latin America, with countries such as Peru, Bolivia, and Chile reporting the highest rates. These trends may be attributed to region-specific environmental or occupational risk factors, genetic predispositions, or improved diagnostic infrastructure (32, 33). In contrast, regions such as Eastern Europe and parts of Sub-Saharan Africa reported the lowest ASIRs, which may reflect underdiagnosis, limited healthcare access, or insufficient disease awareness (34, 35). The variation in EAPC further highlights divergent regional trajectories. For instance, significant increases in ASIR in Ecuador and Kazakhstan can likely be attributed to enhanced diagnostic capabilities and heightened disease recognition, while declining ASIR trends in Ukraine and Belarus may be related to underreporting and ongoing systemic healthcare challenges (16, 36, 37).

Consistent with incidence burden, from 2000 to 2010, the steady rise in mortality and DALYs also reflects the growing recognition and diagnosis of this disease, however, the sharper increase in mortality during this period suggests that improvements in disease detection were not yet matched by advancements in treatment or management strategies, particularly for severe cases. In the US, ILDs and pulmonary sarcoidosis mortality rates doubled from 1980 to 2014, with significant increases in 86.1% of counties, particularly in New England, while smaller rises occurred in Alaska, Nevada, southern Florida, and near the US-Mexico border (38). ILDs mortality rates have significantly increased from 1979 to 2021 in the US, with higher rates observed in males compared to females and in whites compared to blacks, regardless of sex or ethnicity (39). However, in our study, the ASMR for this disease showed a noticeable deceleration in its growth starting from 2014, as indicated by the decrease in the APC from 1.37% (2004–2014) to 0.51% (2014–2019). This trend coincides with the introduction of antifibrotic agents, such as nintedanib and pirfenidone, in 2014, which aim to slow the progression of lung fibrosis, although their widespread adoption has remained limited (40–42). However, growing evidence underscores the benefits of these treatments, providing hope for improved outcomes in ILD management in the future (43).

Aging, a complex process involving mechanisms like genomic instability, cellular senescence, and mitochondrial dysfunction, drives IPF and contributes to the progression of other ILDs, including chronic hypersensitivity pneumonitis and RA-ILD (44). Aging is a natural biological process marked by the gradual decline in functional capacity and a diminished ability to adapt to environmental stimuli (4). Aging significantly impacts lung physiology and contributes to the development and progression of chronic respiratory diseases, including ILDs, through mechanisms such as oxidative stress, telomere attrition, genomic instability, and cellular senescence. These aging-related processes, particularly the senescence-associated secretory phenotype, drive chronic inflammation, tissue dysfunction, and fibrosis, highlighting the need for targeted therapeutic strategies to mitigate their effects (45). In this study, the disease burden is predominantly concentrated in older populations, particularly those aged 50 years and above, with the highest rates observed in individuals aged 75 years and older, underscoring the impact of global population aging. Our APC analysis revealed distinct patterns in the burden of ILDs and pulmonary sarcoidosis. The age effect demonstrated that older adults, particularly those aged 75 years and above, bear the highest burden of disease, consistent with the progressive and chronic nature of ILDs. Furthermore, a consistent gender disparity is observed, with males exhibiting higher incidence, mortality, and DALY rates than females, particularly in middle-aged and older groups, potentially due to biological, occupational, and lifestyle factors.

Several limitations of this study should be noted. First, the classification and diagnosis of ILDs and pulmonary sarcoidosis remain complex due to the diverse nature of the disease spectrum, which can result in variability in epidemiological estimates. Second, the data used in the GBD study were predominantly sourced from hospital records and insurance claims in many regions. This reliance may lead to underrepresentation of cases in low-SDI regions, where healthcare access and data reporting systems are often limited, despite efforts to adjust for such gaps through modeling. Third, the projections are based on historical data, which may limit their ability to fully account for future changes in diagnostic techniques, therapeutic advancements, or evolving environmental factors. Lastly, the lack of detailed data on specific ILD subtypes limits the ability to draw definitive conclusions about individual diseases within the broader ILD spectrum.

## Conclusion

The global burden of ILDs and pulmonary sarcoidosis remains substantial, with notable disparities across age groups, genders, and SDI regions, despite advancements in diagnostics and treatment improving outcomes in recent decades. Understanding these epidemiological patterns is crucial for developing effective prevention and management strategies.

## Data Availability

Publicly available datasets were analyzed in this study. This data can be found here: http://ghdx.healthdata.org/gbd-results-tool.

## Supplementary material

Supplementary Figure 1 Trends in mortality of interstitial lung diseases (ILDs) and pulmonary sarcoidosis by age and sex, 2000–2021. **(A)** Mortality cases. **(B)** Age-standardized mortality rates (ASMR). **(C)** Mortality cases and ASMR stratified by age and sex.

Supplementary Figure 2 Trends in disability-adjusted life years (DALYs) of interstitial lung diseases (ILDs) and pulmonary sarcoidosis by age and sex, 2000–2021. **(A)** DALYs cases. **(B)** Age-standardized DALYs rates (ASDR). **(C)** DALYs cases and ASDR stratified by age and sex.

Supplementary Table 1 Mortality of interstitial lung disease and pulmonary sarcoidosis between 2000 and 2021.

Supplementary Table 2 DALYs of interstitial lung diseases and pulmonary sarcoidosis between 2000 and 2021.

Supplementary Table 3 Incidence of interstitial lung diseases and pulmonary sarcoidosis between 2000 and 2021 at national levels.

Supplementary Table 4 Mortality of interstitial lung diseases and pulmonary sarcoidosis between 2000 and 2021 at national levels.

Supplementary Table 5 DALYs of interstitial lung diseases and pulmonary sarcoidosis between 2000 and 2021 at national levels.
