# Supplementary figures and images for: Global, regional, and national burden of interstitial lung diseases and pulmonary sarcoidosis from 2000 to 2021: a systematic analysis of incidence, mortality, and disability-adjusted life years
Source: Front Public Health. 2025 Jun 16;13:1578480. doi: 10.3389/fpubh.2025.1578480 (PMC12206815; doi:10.3389/fpubh.2025.1578480)

A

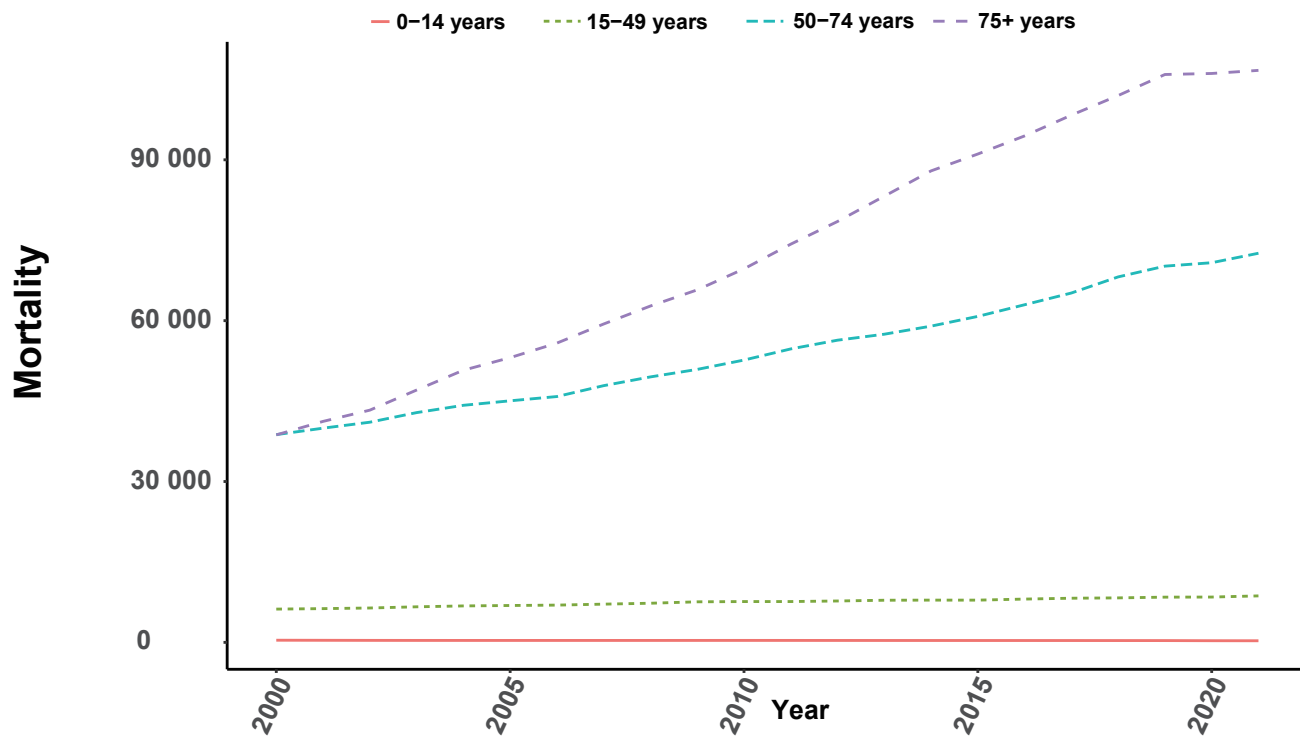

B

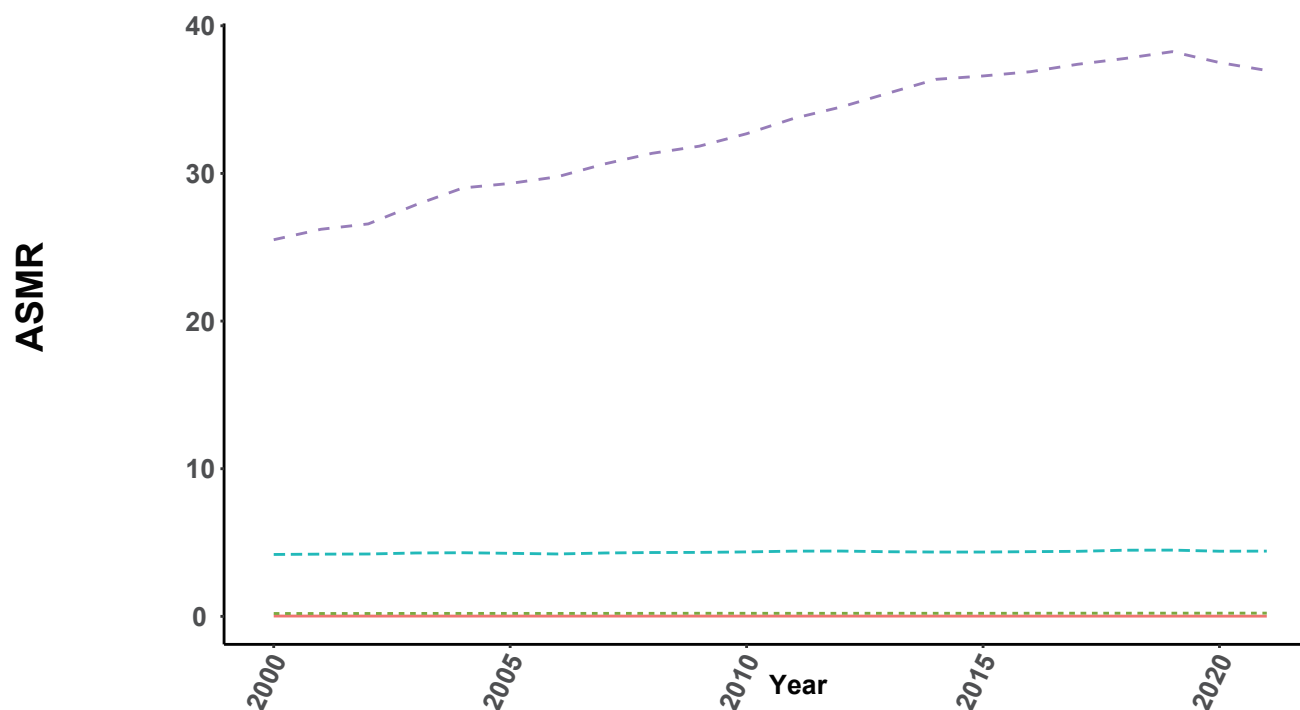

C

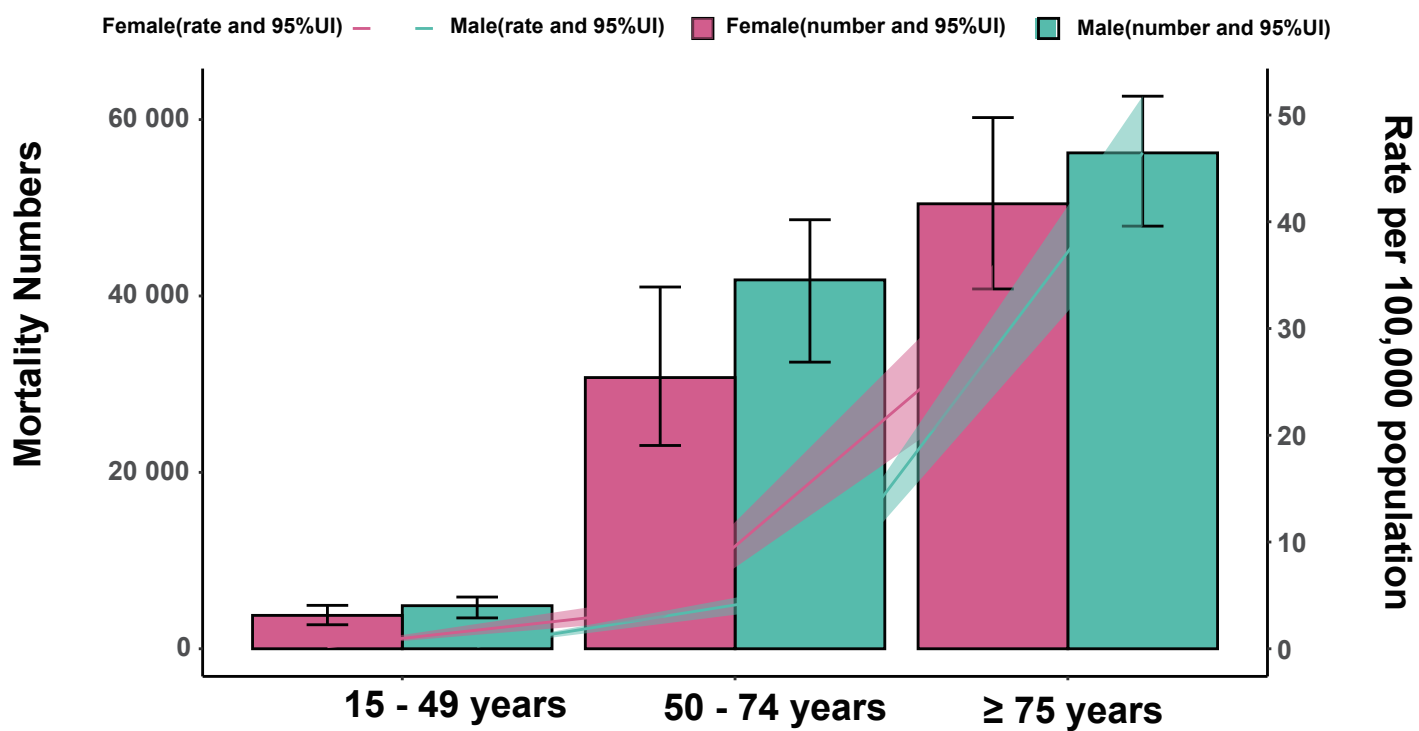

Supplement: Supplementary Figure 1 — Trends in mortality of interstitial lung diseases (ILDs) and pulmonary sarcoidosis by age and sex, 2000–2021. (A) Mortality cases. (B) Age-standardized mortality rates (ASMR). (C) Mortality cases and ASMR stratified by age and sex. [file Image_1.pdf]

**A**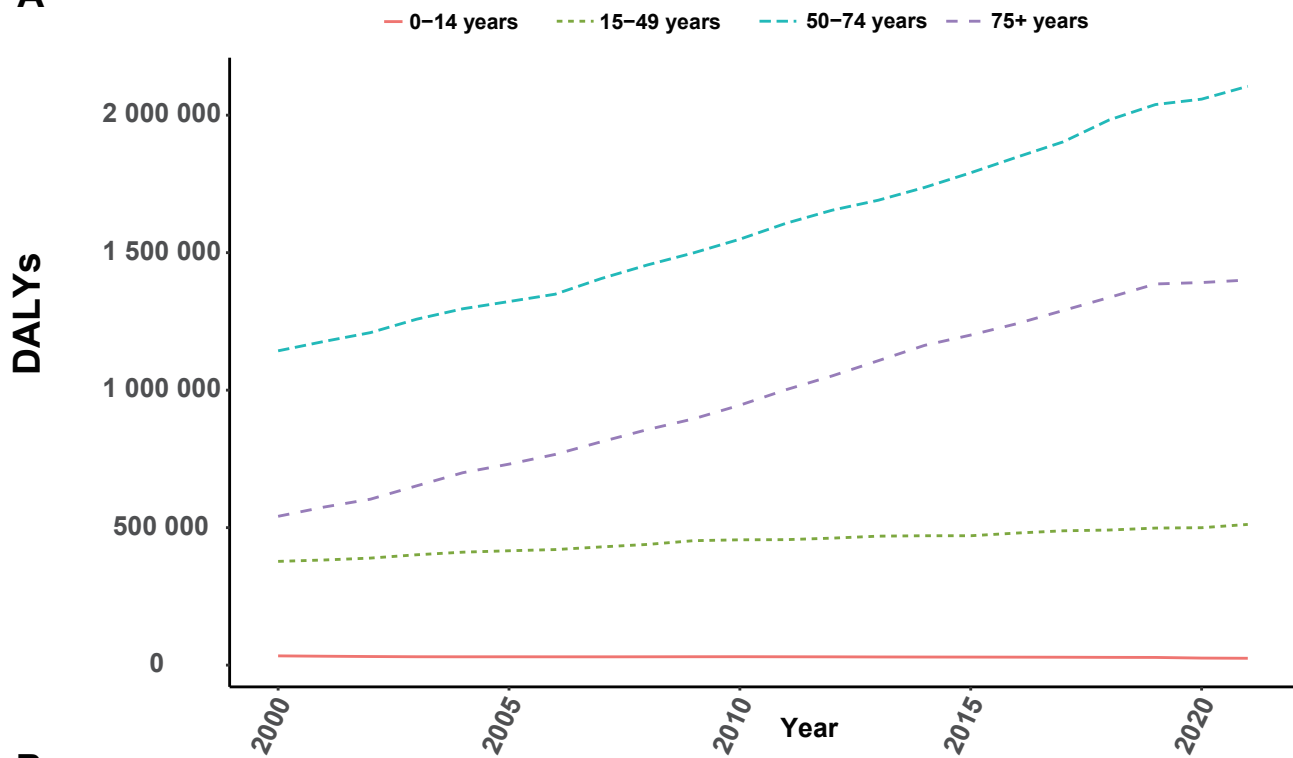**B**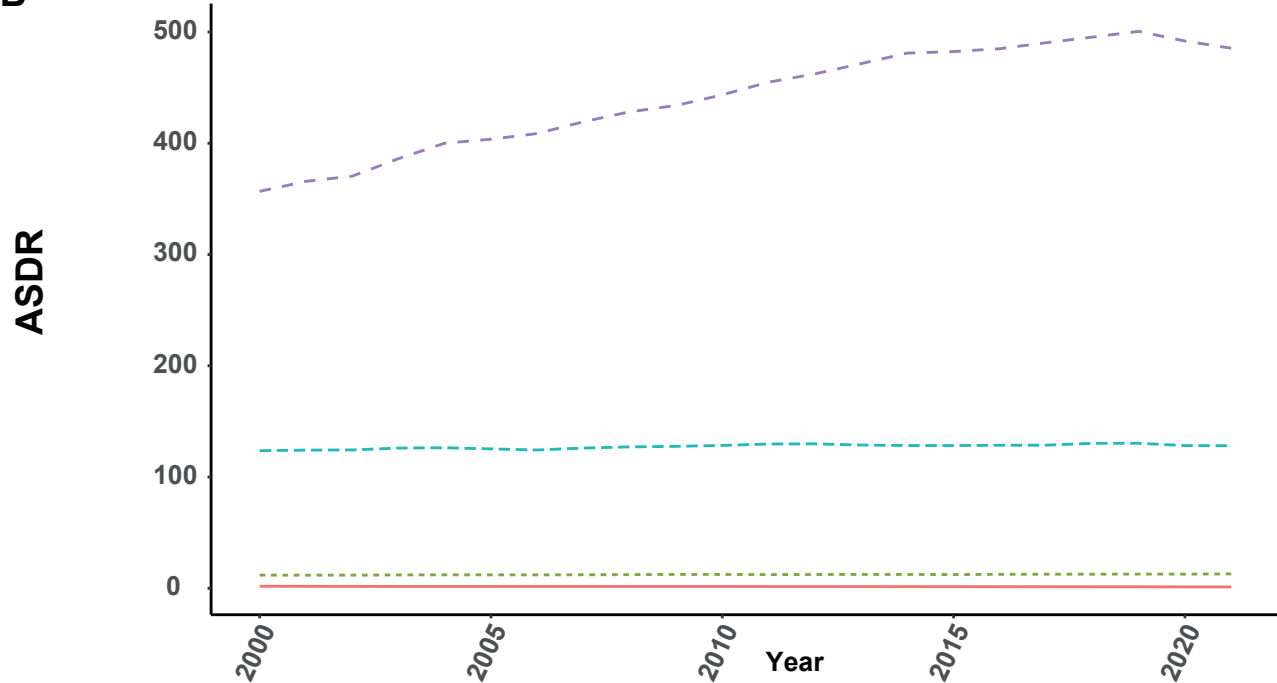**C**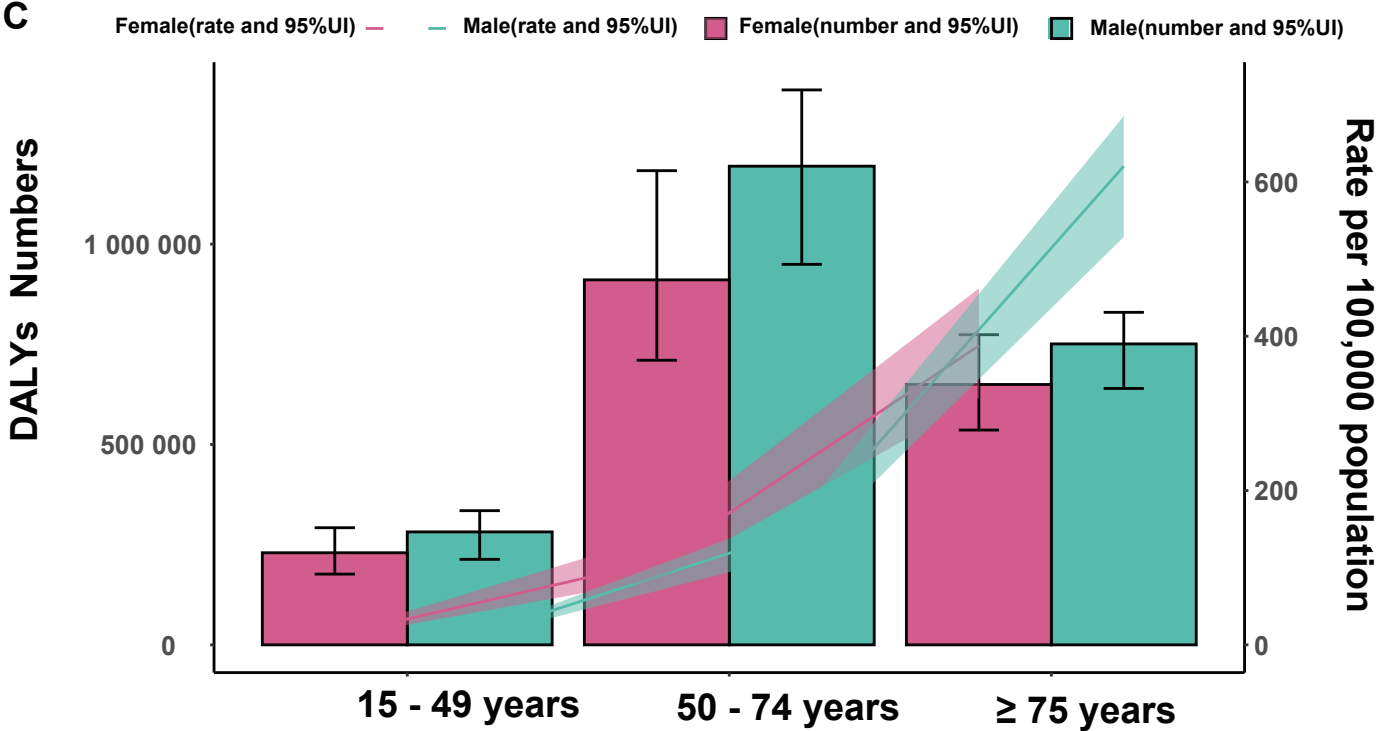

Supplement: Supplementary Figure 2 — Trends in disability-adjusted life years (DALYs) of interstitial lung diseases (ILDs) and pulmonary sarcoidosis by age and sex, 2000–2021. (A) DALYs cases. (B) Age-standardized DALYs rates (ASDR). (C) DALYs cases and ASDR stratified by age and sex. [file Image_2.pdf]
